# Supplementary material for: Slit2 as a β-catenin/Ctnnb1-dependent retrograde signal for presynaptic differentiation
Source: eLife. 2015 Jul 10;4:e07266. doi: 10.7554/eLife.07266 (PMC4498096; doi:10.7554/eLife.07266)
Supplement: Supplementary file 1. — The sequences of primers used for qRT-PCR. DOI: http://dx.doi.org/10.7554/eLife.07266.015 [file elife07266s001.docx]

**Supplementary File 1. The sequences of primers used for qRT-PCR**

| Gene name | Primers (5’-3’) |
| --- | --- |
| *C-Myc* | ATGCCCCTCAACGTGAACTTC / GTCGCAGATGAAATAGGGCTG |
| *Axin2* | ATGAGTAGCGCCGTGTTAGTG / GGGCATAGGTTTGGTGGACT |
| *Lamb1* | AGACCCGAAGAAAAGACAGGC / CCATAGGGCTAGGACACCAAA |
| *Lamb2* | CCCCGTCCTTGGATGTACCT / CAGTAGGGTTGAGGGCTATGC |
| *Lama1* | CAGCGCCAATGCTACCTGT / AGGATTCGTACTGTTACCGTCA |
| *Lama2* | TCCCAAGCGCATCAACAGAG / CAGTACATCTCGGGTCCTTTTTC |
| *Col9a1* | CGACCGACCAGCACATCAA / AGGGGGACCCTTAATGCCT |
| *Col13a1* | AATGGAAGTTCTACTCGCGTAGG / TTCTCGCCTGGTTGACCTTTG |
| *Mmp9* | GCAGAGGCATACTTGTACCG / TGATGTTATGATGGTCCCACTTG |
| *Mmp13* | TGTTTGCAGAGCACTACTTGAA / CAGTCACCTCTAAGCCAAAGAA |
| *Itga3* | CCTCTTCGGCTACTCGGTC / CCAGTCCGGTTGGTATAGTCATC |
| *Itgb1* | ATGCCAAATCTTGCGGAGAAT / TTTGCTGCGATTGGTGACATT |
| *Vegf* | CTGCCGTCCGATTGAGACC / CCCCTCCTTGTACCACTGTC |
| *Reelin* | GTACCGGGACAGGAATACCAT / AAGCTGAGGTTGGTTGTAGGC |
| *Nrg3* | GGGAGTTACGCTGTAGCGAC / GCCTACCACGATCCATTTAAGC |
| *Cdnf* | CCTTTTGCGCCGGGTTTTG / AGGGAGTTGTAGAATCGGTCTAA |
| *Nt4* | TGAGCTGGCAGTATGCGAC / CAGCGCGTCTCGAAGAAGT |
| *Hgf* | ACTTCTGCCGGTCCTGTTG / CCCCTGTTCCTGATACACCT |
| *Gdnf* | GCCGGACGGGACTCTAAGAT / CGTCATCAAACTGGTCAGGATAA |
| *NT3* | AGTTTGCCGGAAGACTCTCTC/ GGGTGCTCTGGTAATTTTCCTTA |
| *Fgf7* | TGGGCACTATATCTCTAGCTTGC / GGGTGCGACAGAACAGTCT |
| *Fgf10* | TCAGCGGGACCAAGAATGAAG / CGGCAACAACTCCGATTTCC |
| *Fgf22* | CTCTGTGGACTGTAGGTTCCG / GAGGCGTATGTGTTGTAGCC |
| *Wnt3A* | CTCCTCTCGGATACCTCTTAGTG CCAAGGACCACCAGATCGG |
| *Wnt11* | GCACTGAATCAGACGCAACAC / CGACAGGGCATACACGAAGG |
| *Wnt9A* | GGCCCAAGCACACTACAAG / AGAAGAGATGGCGTAGAGGAAA |
| *Bmp2* | GGGACCCGCTGTCTTCTAGT / TCAACTCAAATTCGCTGAGGAC |
| *Bmp4* | ATTCCTGGTAACCGAATGCTG / CCGGTCTCAGGTATCAAACTAGC |
| *Bmp5* | TTACTTAGGGGTATTGTGGGCT / TGAACGTGATTGTCTCCCAAG |
| *Bmp6* | GCGGGAGATGCAAAAGGAGAT / ATTGGACAGGGCGTTGTAGAG |
| *Bmp7* | CCTGTCCATCTTAGGGTTGCC / GGCCTTGTAGGGGTAGGAGA |
| *Bmp9* | CCCTGGGATTGTCTGGAGC / AGGTTAAGGCTGCGTAGGAAA |
| *EphrinA3* | AGGTGAACGTGAACGACTATCT / GCGCTGTAACGCTGGAACT |
| *Sema3b* | TAGCAGGGCTAGGGGATACTG / CCTGAAGGTTCGGACACCA |
| *Sema3c* | GCCAGCATCAACAATCAAAGTT / TCTGAATCACCCGGACGAAAT |
| *Sema4c* | GAGATGTGGTGGAACCTTGTG/CAGGGTCAGTGTCAGGAAGTC |
| *Sema7a* | CACCGTGCTTTTCCATGAGC / CGGGGAAGTTGAAGTGGTAGAC |
| *Sema5a* | GACTTGCTAGGCCCGAGAC / TCTGAACTCCCGTAACCAGGG |
| *Netrin* | CAGCCTGATCCTTGCTCGG / GCGGGTTATTGAGGTCGGTG |
| *Slit1* | GAACTCAACGGCAACAACATC / TCAGGCAACACTTGTAGCTGG |
| *Slit2* | GGCAGACACTGTCCCTATCG / ATCTATCTTCGTGATCCTCGTGA |
| *Slit3* | TGCCCCACCAAGTGTACCT / CGCCTCTCTCGATGATGCT |
| *Cxcl2* | CCAACCACCAGGCTACAGG / GCGTCACACTCAAGCTCTG |
| *Cxcl15* | TCGAGACCATTTACTGCAACAG / CATTGCCGGTGGAAATTCCTT |
| *Unc5b* | CGGGACGCTACTTGACTCC / GGTGGCTTTTAGGGTCGTTTAG |
| *Unc5c* | CTGCGGACTGGGACTAGGATA / GGTTTCTGGGAGTTCGTGAAAA |
